# Supplementary material for: Resting-state electroencephalography and magnetoencephalography in migraine–a systematic review and meta-analysis
Source: J Headache Pain. 2024 Sep 11;25(1):147. doi: 10.1186/s10194-024-01857-5 (PMC11389598; doi:10.1186/s10194-024-01857-5)
Supplement: Supplementary file 1 — Supplementary Material 1 [file 10194_2024_1857_MOESM1_ESM.docx]

Supplementary Material

**Fig. S1** Funnel plots and Egger’s Test results for publication bias of meta-analyses


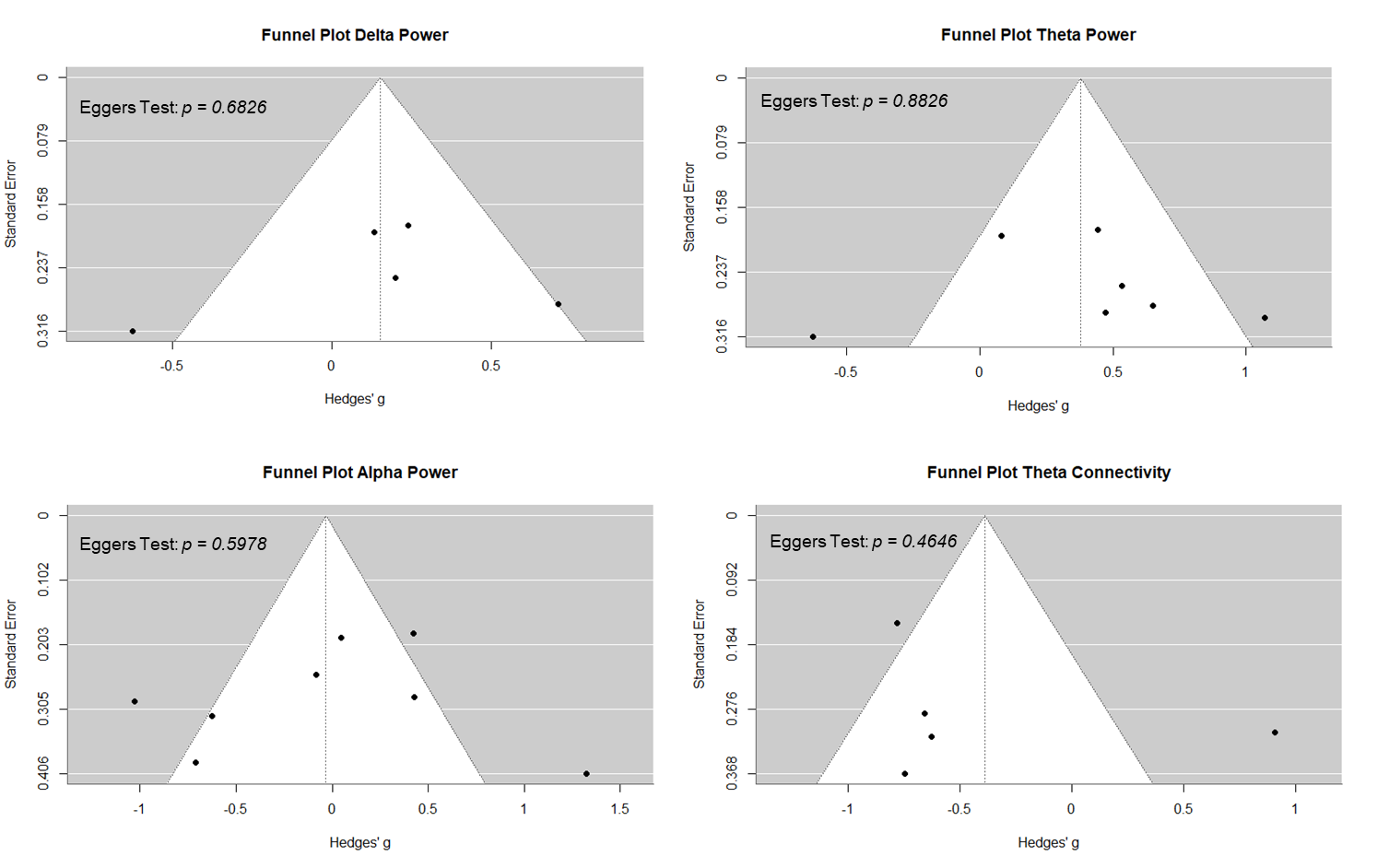


**Fig. S2** Effect direction plot for comparing PAF, oscillatory power, or connectivity between episodic and chronic migraine


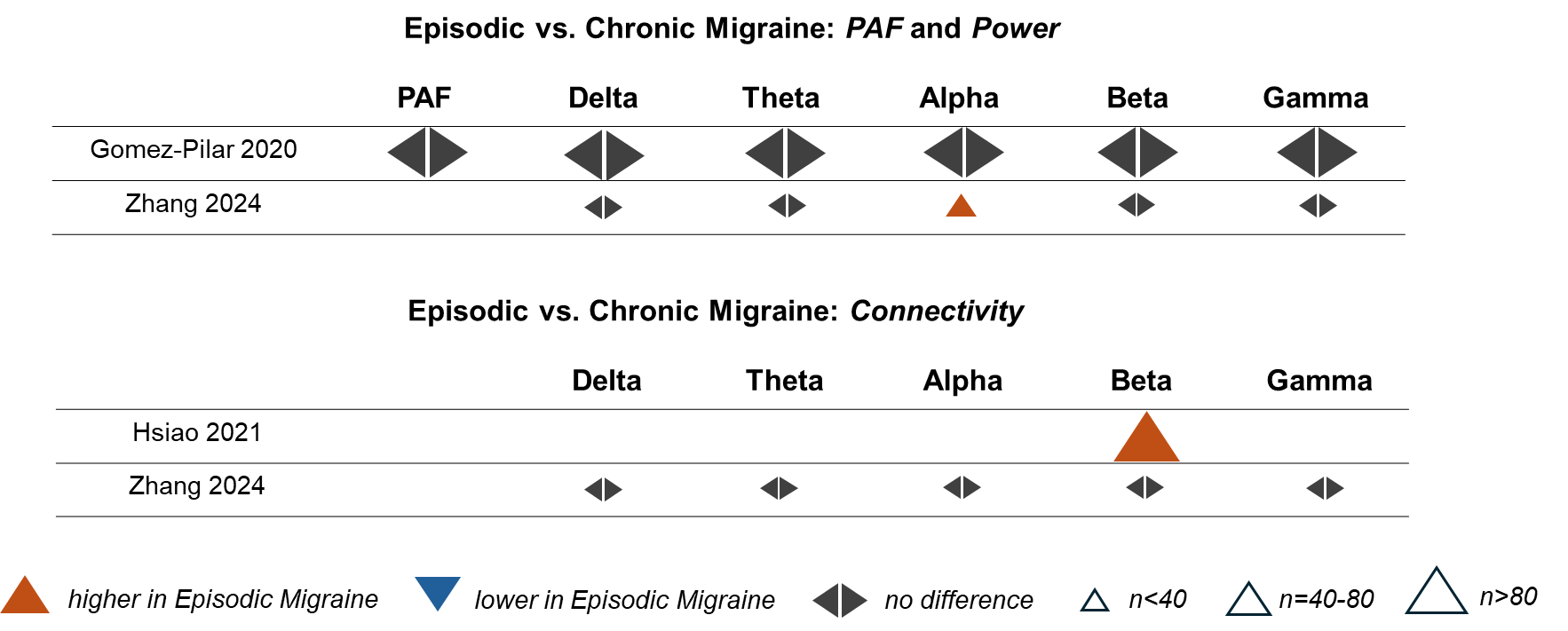


**Fig. S3** Effect direction plot for comparing PAF, oscillatory power, or connectivity between migraine with and without aura


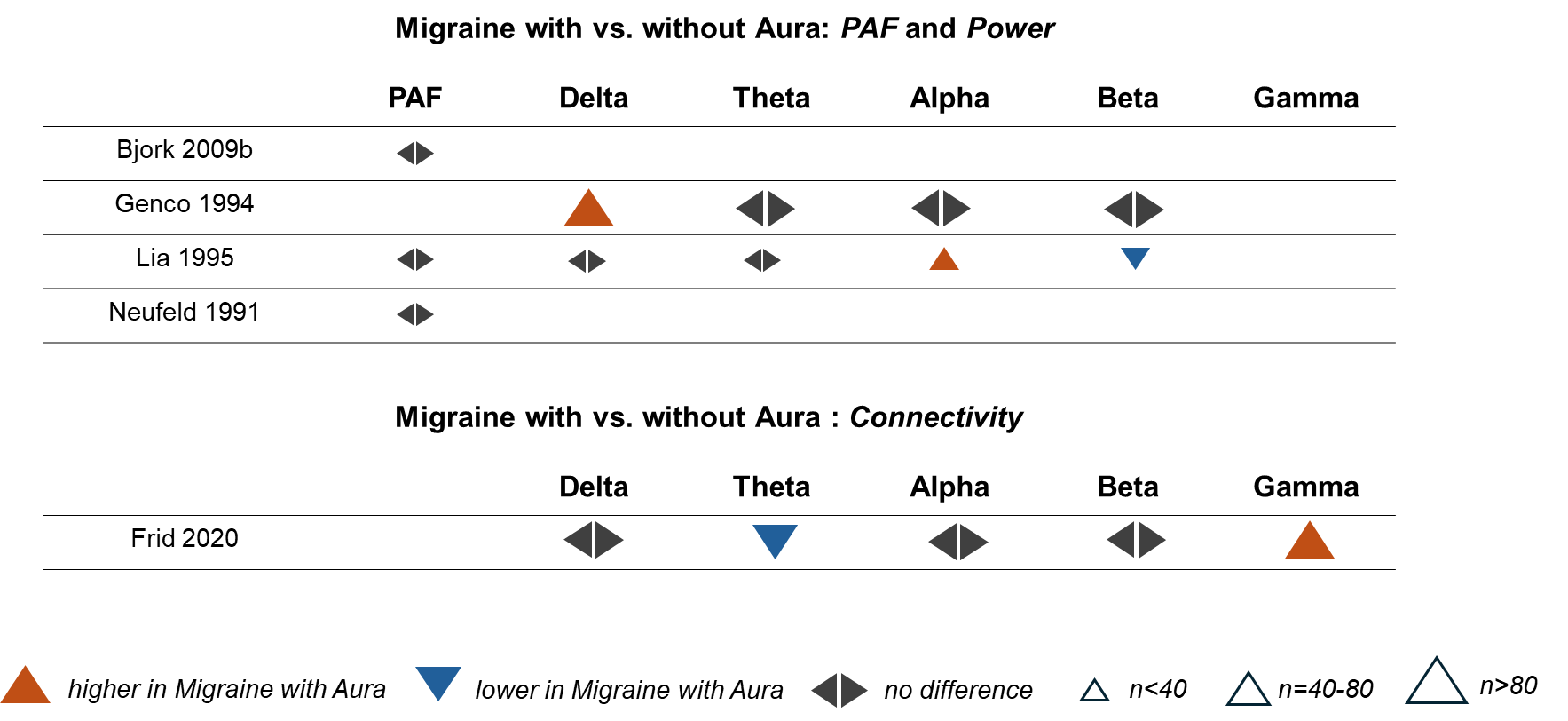


**Fig. S4** Sample size of individual studies


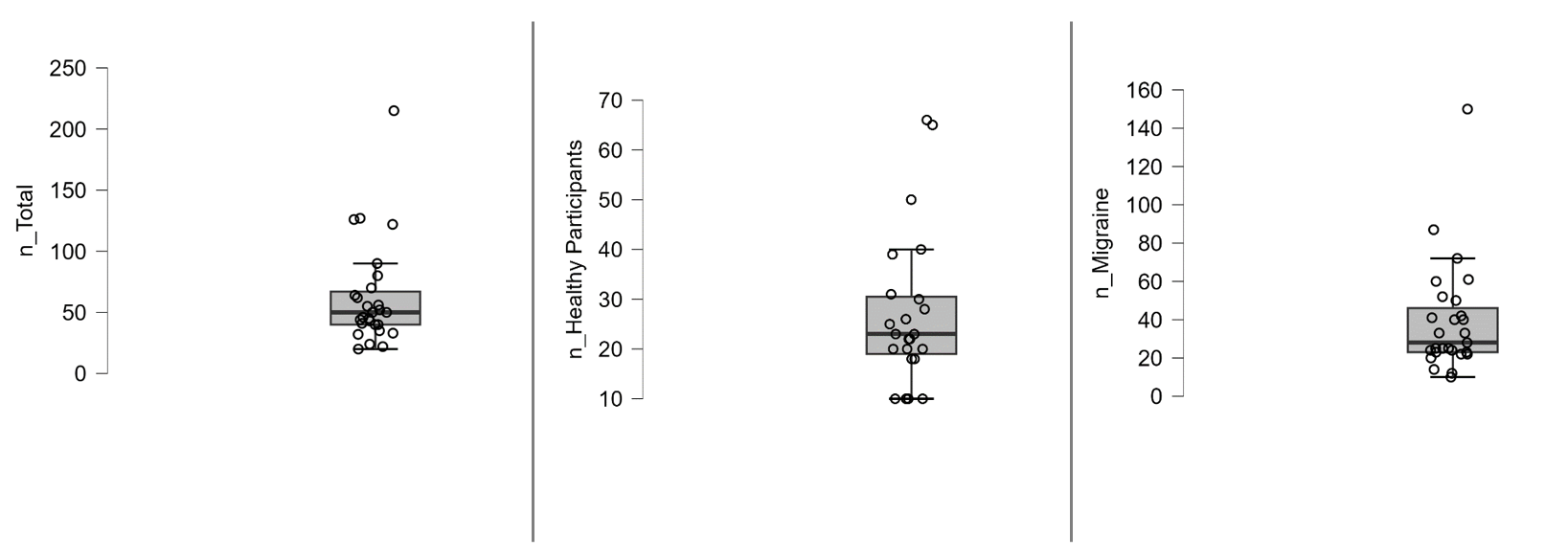


**Table S1** Individual scoring of Risk of Bias Assessment for included studies

| **Study ID** | **Title** | **Selection: Case Definition** | **Selection: Representativeness** | **Selection: Controls** | **Selection:**  **Definition of Controls** | **Comparability: Anxiety/Depression** | **Comparability: Any other factor** | **Outcome: Independent EEG preprocessing**  **/analysis** | **Outcome: Description of EEG measurement** | **Outcome: Statistical test** |
| --- | --- | --- | --- | --- | --- | --- | --- | --- | --- | --- |
| Bjørk 2008 | Quantitative EEG power and asymmetry increase 36 h before a migraine attack. | Low RoB | Low RoB |  |  | High RoB | Low RoB | High RoB | Low RoB | Low RoB |
| Bjørk 2009 b | The occipital alpha rhythm related to the "migraine cycle" and headache burden: a blinded, controlled longitudinal study. | Low RoB | Low RoB | Low RoB | Low RoB | High RoB | Low RoB | High RoB | Low RoB | Low RoB |
| Bjørk 2009 a | Interictal quantitative EEG in migraine: a blinded controlled study. | Low RoB | Low RoB | Low RoB | Low RoB | High RoB | Low RoB | High RoB | Low RoB | Low RoB |
| Cao 2016 | Resting-state EEG power and coherence vary between migraine phases. | Low RoB | Low RoB | Low RoB | Low RoB | Low RoB | Low RoB | High RoB | Low RoB | High RoB |
| Cao 2018 | Exploring resting-state EEG complexity before migraine attacks. | Low RoB | High RoB | Low RoB | Low RoB | Low RoB | Low RoB | Low RoB | Low RoB | Low RoB |
| Chamanzar 2021 | Abnormalities in cortical pattern of coherence in migraine detected using ultra high-density EEG. | Low RoB | High RoB | Low RoB | Low RoB | High RoB | Low RoB | High RoB | Low RoB | Low RoB |
| deTommaso 1998 | EEG spectral analysis in migraine without aura attacks. | Low RoB | Low RoB | Low RoB | Low RoB | High RoB | Low RoB | High RoB | Low RoB | Low RoB |
| Frid 2020 | A Biomarker for Discriminating Between Migraine With and Without Aura: Machine Learning on Functional Connectivity on Resting-State EEGs. | Low RoB | High RoB |  |  | High RoB | Low RoB | Low RoB | Low RoB | Low RoB |
| Genco 1994 | EEG features in juvenile migraine: topographic analysis of spontaneous and visual evoked brain electrical activity: a comparison with adult migraine. | Low RoB | High RoB | Low RoB | High RoB | High RoB | Low RoB | High RoB | High RoB | High RoB |
| Gomez-Pilar 2020 | Exploring EEG Spectral Patterns in Episodic and Chronic Migraine During the Interictal State: Determining Frequencies of Interest in the Resting State. | Low RoB | Low RoB | Low RoB | High RoB | Low RoB | Low RoB | High RoB | Low RoB | Low RoB |
| Hsiao 2021 | Migraine chronification is associated with beta-band connectivity within the pain-related cortical regions: a magnetoencephalographic study. | Low RoB | High RoB | Low RoB | Low RoB | Low RoB | Low RoB | Low RoB | Low RoB | Low RoB |
| Kim 2023 | Quantitative electroencephalography as a potential biomarker in migraine. | Low RoB | Low RoB |  |  | High RoB | High RoB | High RoB | Low RoB | Low RoB |
| Lei 2023 | Resting-state electroencephalography microstate dynamics altered in patients with migraine with and without aura-A pilot study. | Low RoB | Low RoB | Low RoB | Low RoB | High RoB | Low RoB | High RoB | Low RoB | Low RoB |
| Li 2016 | Abnormal resting-state brain activity in headache-free migraine patients: A magnetoencephalography study. | Low RoB | High RoB | Low RoB | High RoB | High RoB | Low RoB | Low RoB | Low RoB | Low RoB |
| Li 2022 | Abnormalities in resting-state EEG microstates are a vulnerability marker of migraine. | Low RoB | High RoB | Low RoB | Low RoB | Low RoB | Low RoB | High RoB | Low RoB | Low RoB |
| LIA 1995 | COMPUTERIZED EEG-ANALYSIS IN MIGRAINE PATIENTS | Low RoB | High RoB | High RoB | Low RoB | High RoB | Low RoB | High RoB | Low RoB | Low RoB |
| Liu 2015 | Resting state brain activity in patients with migraine: a magnetoencephalography study. | Low RoB | High RoB | Low RoB | Low RoB | High RoB | Low RoB | High RoB | Low RoB | Low RoB |
| Martins 2020 | Brain state monitoring for the future prediction of migraine attacks. | Low RoB | High RoB |  |  | High RoB | Low RoB | High RoB | Low RoB | Low RoB |
| Neufeld 1991 | EEG and topographic frequency analysis in common and classic migraine. | Low RoB | Low RoB | High RoB | High RoB | High RoB | Low RoB | High RoB | Low RoB | High RoB |
| O'Hare 2024 | No Evidence of Cross-Orientation Suppression Differences in Migraine with Aura Compared to Healthy Controls. | High RoB | Low RoB | High RoB | High RoB | High RoB | Low RoB | Low RoB | Low RoB | Low RoB |
| Ojha 2024 | Resting-state Quantitative EEG Spectral Patterns in Migraine During Ictal Phase Reveal Deviant Brain Oscillations: <i>Potential Role of Density Spectral Array</i> | Low RoB | Low RoB | Low RoB | Low RoB | Low RoB | Low RoB | High RoB | Low RoB | Low RoB |
| Pan 2022 | Resting-state occipital alpha power is associated with treatment outcome in patients with chronic migraine. | Low RoB | Low RoB | Low RoB | Low RoB | Low RoB | Low RoB | High RoB | Low RoB | Low RoB |
| Pelayo-González 2023 | Quantitative Electroencephalographic Analysis in Women with Migraine during the Luteal Phase | Low RoB | Low RoB | Low RoB | Low RoB | Low RoB | High RoB | Low RoB | Low RoB | Low RoB |
| Rocha 2021 | Could cathodal transcranial direct current stimulation modulate the power spectral density of alpha-band in migrainous occipital lobe? | Low RoB | Low RoB | Low RoB | High RoB | High RoB | Low RoB | High RoB | Low RoB | Low RoB |
| Wu 2016 | Multi-frequency analysis of brain connectivity networks in migraineurs: a magnetoencephalography study. | Low RoB | High RoB | Low RoB | High RoB | High RoB | Low RoB | High RoB | Low RoB | Low RoB |
| Zhang 2024 | Altered Neuromagnetic Activity in the Default Mode Network in Migraine and Its Subgroups (Episodic Migraine and Chronic Migraine). | Low RoB | Low RoB | Low RoB | High RoB | Low RoB | Low RoB | High RoB | Low RoB | Low RoB |
| Zhou 2023 | Spatio-temporal dynamics of resting-state brain networks are associated with migraine disability. | Low RoB | Low RoB | Low RoB | Low RoB | Low RoB | Low RoB | Low RoB | Low RoB | Low RoB |

**S1** Newcastle-Ottawa quality assessment scale adapted for cross-sectional/ case-control studies. Adaption for M/EEG studies in Migraine

Note: A study can be awarded a maximum of one star for each numbered item within the Selection

and Exposure categories. A maximum of two stars can be given for Comparability. Items are rated as *high* (indicating negative study quality) or *low* (indicating positive study quality) Risk of Bias.

**Selection (Maximum 4 stars)**

1) Is the case definition (migraine) adequate?

a) yes, with independent validation (eg. self reported doctor’s diagnosis, reference to primary medical record source,

international criteria) **Low ROB**

b) no, based on self reports

c) no description

2) Representativeness of the cases

a) Truly representative of the average in the target population. (all subjects or random sampling) **Low ROB**

b) Somewhat representative of the average in the target population. (non-random sampling) **Low ROB**

c) Selected group of users

d) No description of the sampling strategy

3) Matching/Selection of Controls

a) Matched by sex and/or age **Low ROB**

b) Matched by other factor

c) No description

4) Definition of Controls

a) No history of migraine and no current severe neurological or psychiatric diagnosis **Low ROB**

b) No description of source

**Comparability (Maximum 2 stars)**

1) Comparability of cases and controls on the basis of the design or analysis

a) The study controls for the most important factor (anxiety/depression). **Low ROB**

b) The study controls for any additional factor (eg. age, medication usage, duration of migraine) **Low ROB**

**Outcome (Maximum 3 stars)**

1) Assessment of outcome (EEG findings)

a) Independent blind assessment **Low ROB**

b) Clear description of method for EEG data acquisition (number and placement of electrodes, equipment, sample

rating) and data processing (how the parameters were extracted from the EEG data) **Low ROB**

c) No description of method for EEG data acquisition and/or data processing

2) Statistical test

a) The statistical test used to analyze the data is clearly described and appropriate, and the measurement of the

association is presented, including confidence intervals/effect size and the probability level (p value). **Low ROB**

b) The statistical test is not appropriate, not described or incomplete.

**S2** Search Strings

**MEDLINE, PubMed Central and Bookshelf (via PubMed)**

migrain* [Title/Abstract] AND (qEEG [Title/Abstract] OR EEG [Title/Abstract] OR electroencephalogr* [Title/Abstract] OR MEG [Title/Abstract] OR magnetoencephalogr*)

**Web of Science Core Collection (via Web of Science)**

migrain* AND (qEEG OR EEG OR electroencephalogr* OR MEG OR magnetoencephalogr*)

+Topic (Abstract/Title)

**EMBASE (via OVID)**

(migrain* AND (qEEG OR EEG OR electroencephalogr* OR MEG OR magnetoencephalogr*)).ab,ti.

**S3** Code (R Version 4.1.2)

**Calculation of hedges g for MWU-Tests**

library(esc)

# Given p-value

p_value <- 0.xyz

# Given total N (n1+2)

n1 <- x

n2 <- y

N <- x+y

# Calculate the z-score

z_score <- qnorm(p_value / 2, lower.tail = FALSE)

# Calculate eta-squared

eta_squared <- (z_score^2) / (N)

# convert to cohens d based on doi:10.1037/a0024338

d <- (2*sqrt(eta_squared))/(sqrt(1-eta_squared))

# convert cohens d to hedges g

totaln <- N

hedges_g(d, totaln)

**RE-Meta-Analysis**

library(metafor)

library(readxl)

# Specify the path to your Excel file

file_path <- read_excel("Path")

# Load the data from the Excel file

data <- read_excel("Path")

# Calculate the variances for Hedges' g

data$var_g <- (data$n1 + data$n2) / (data$n1 * data$n2) + (data$hedges_g^2 / (2 * (data$n1 + data$n2)))

# Assign study labels to studies and data

study = c("Study1”)

study_labels <-data$study

# Conduct the meta-analysis

res <- rma(yi = hedges_g, vi = var_g, data = data, method = "REML")

# Print the results

print(res)

**Create a forest plot**

forest(res, slab = study_labels, xlab = "Effect Size (Hedges' g)")

**Create a funnel plot**

funnel(res, main="Funnel Plot Theta Power", xlab="Hedges' g", ylab="Standard Error")

**Conduct Egger’s test**

egger_test <- regtest(res, model = "lm")

print(egger_test)
